# Supplementary material for: Breeding D1-Type Hybrid Japonica Rice in Diverse Upland Rainfed Environments
Source: Int J Mol Sci. 2025 Mar 31;26(7):3246. doi: 10.3390/ijms26073246 (PMC11989851; doi:10.3390/ijms26073246)
Supplement: Supplementary file 1 [file ijms-26-03246-s001.zip › Finally-Supplementary Materials-IJMS3515233/Supplementary Materials-Table S1-S3,TableS7 ,FigS1-S2-Revised.pdf]

Supplementary Materials

Table S1-1. Grain quality of 'DHY615' in paddy field and rainfed upland planting in different ecological regions.

Regions	Brown rice (%)	Milled rice (%)	Ratio of length/ width	Chalk grain (%)	Chalk degree (%)	Amylose content (%)	Gel consistency (mm)	Grain quality
Lufeng, Yunnan	84.10	68.80	1.90	14.00	1.70	16.40	64.00	III
Lueyang, Shanxi	83.80	71.40	1.90	12.00	1.70	16.50	70.00	II
Huishui, Guizhou	82.50	67.00	1.90	9.00	1.50	17.20	64.00	III
Anshi, Hubei	82.60	63.00	2.00	13.00	0.90	18.40	84.00	III
Yanshang,Yunnan*	83.60	68.10	2.00	9.00	1.20	15.40	74.00	II

Note: Data from national field yield trials in 2020-2022, * rainfed upland planting regions. There are three levels of high-quality indica rice, with I-the highest level, II-better level, and III-good level.

Table S1-2. Grain quality of 'DHY34' in paddy field and rainfed upland planting in Yunnan Province.

Regions	Brown rice (%)	Milled rice (%)	Ratio of length/ width	Chalk grain (%)	Chalk degree (%)	Amylose content (%)	Gel consistency (mm)	Grain quality (grade)
Longyang, Baoshan	81.00	62.60	2.2	30.00	3.00	15.80	70.00	III
Jingyuan, Kunming	83.90	67.80	1.9	19.00	2.30	15.30	72.00	II
Wenhua,Jingdong *	84.10	65.00	2.1	5.00	0.60	17.20	72.00	III
Manwang,Jingdong*	83.60	65.90	2.1	5.00	0.40	17.60	76.00	III

Note: Data from field yield trials in 2020, * rainfed upland planting regions. There are three levels of high-quality indica rice, with I-the highest level, II-better level, and III-good level.

Table S1-3. Comparative analysis of physical and chemical quality of 'DHY34' in paddy field and rainfed upland planting in Yunnan Province

Regions	Jingyuan, Kunming	Wenhua, Jingdong*	Manwang, Jingdong*	International/ National standard	Synthesized assessment
Protein (g/100g)	7.95	9.54	9.22	/	/
Fat (g/100g)	2.42	2.32	2.15	/	/
Fe (mg/kg)	146.00	43.10	72.00	23.00	Rich
Ca (mg/kg)	264.00	205.00	307.00	130.00	
Zn (mg/kg)	17.95	26.32	24.17	17.00	Rich
K (mg/kg)	2280.00	2310.00	2590.00	1030.00	
Se (mg/kg)	0.06	0.04	0.05	≥ 0.07	Rich
Mg (mg/kg)	1080.00	1130.00	1220.00	340.00	Rich
Mn (mg/kg)	30.40	37.50	40.90	12.90	Rich
Na (mg/kg)	15.00	15.50	14.90	40.00	Na (mg/kg)
Cu (mg/kg)	3.82	5.42	2.06	3.00	Cu (mg/kg)
As (mg/kg)	0.07	0.05	0.02	≤ 0.20/ 0.15	Safety
Pb (mg/kg)	/	0.02	0.03	≤ 0.20	Safety
Cd (mg/kg)	0.01	0.04	0.10	≤ 0.40/ 0.20	Safety

Note: Data from field yield trials in 2020, * rainfed upland planting regions.**Table S2.** Identification and performance of resistance/tolerance to bio-and-abiotic stresses of 'DHY615' in Southwestern China

Regions	Leaf blast disease level	Spike blast disease level	Loss rate disease level	Comprehensive Resistance index of rice blast	Sheath blight resistance	Bacterial blight resistance	Rice false smut	Cold& drought tolerance
Yunnan	3.00	3.00	7.00	5.75	1.00	3.00	1.00	3.00 &1~ 3.00*
Sichuan	3.00	3.00	1.00	1.00	/	/	/	/
Guizhou	3.00	7.00	5.00	5.00	/	/	/	3.00

Note: Data from national field yield trials in 2020-2022. Resistance levels were classified into five grades: 1 (high resistance), 3 (resistance), 5 (moderate resistance), 7 (moderate sensitivity), and 9 (high sensitivity). "/" indicates unavailable data. Disease resistance was assessed following national standards DB42/T 1404-2018 and NY/T 2863-2015. *Adopted from [52].

Table S3. Mean values of yield-related characteristics (per plant) among 'DHY615' and its parent in paddy field and rainfed upland in Yunnan Province.

Traits	H479A (Paddy field)	Nan615 (Paddy field)	DHY615 (Paddy field)	DHY615 (Rainfed upland)
Plant height (cm)	104.20 ± 3.10	82.0 ± 1.70***	105.86 ± 7.02	102.50 ± 4.70
Effective spikes (Ten thousands/mu)	7.60 ± 1.10***	4.60 ± 1.50***	23.70 ± 7.35	23.50 ± 5.66
Panicle length (cm)	21.60 ± 1.60	16.50 ± 1.60*	20.82 ± 2.16	20.50 ± 1.64
No. of grains per panicle	159.50 ± 35.60	135.00 ± 37.30***	161.52 ± 35.89	141.50 ± 30.30
Seed-setting rate (%)	/	80.92 ± 9.70	83.78 ± 10.07	78.80 ± 9.90
Thousand seed weight (g)	/	25.63 ± 1.10	25.43 ± 1.00	25.60 ± 1.12
Whole growth duration (d)	140.00 ± 9.08	160.00 ± 10.23	174.3 ± 10.39	137.30 ± 12.70*

Note: "/" indicates unavailable data ,Asterisks indicate significant differences: *$p < 0.05$, **$p < 0.01$, and ***$p < 0.001$.

Table S7. List of primer sequences used in this study.

Primer name	Primer Sequence (5'–3')	Tm	Primer function	Band size
ESP	TTGTTTGGAGCTTGCTGATG	55℃	test *OsBADH2* gene	Aroma: 580 bp and 255 bp double bands Non-aromatic: 580 bp and 355 bp double bands
IFAP	CATAGGAGCAGCTGAAATATATACC			
INSP	CTGGTAAAAAGATTATGGCTTCA			
EAP	AGTGCTTTACAAAGTCCCGC			
RfMAS-1Fb	CCTCTTTGGATGGAGGGAGTAGTTT	56℃	test *Rf1a* gene	Rf gene: 521 bp
RfMAS-1Rb	ATTGGATTTCAATCGGATTTACGGACG			rf gene: 442 bp

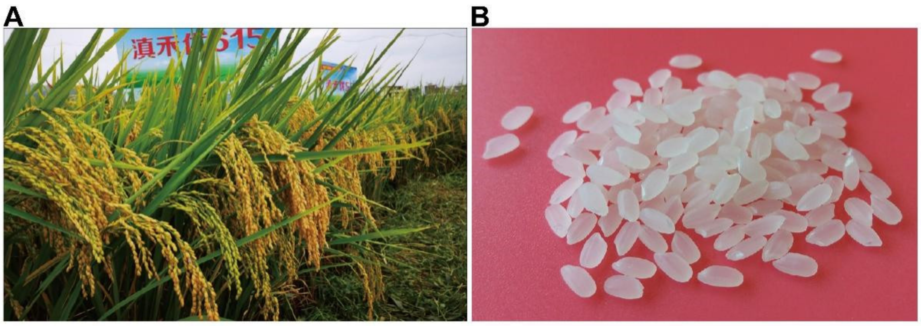

Figure S1. Plant morphology and polished rice grains of 'DHY615'. (**A**) Plant morphology of 'DHY615' in paddy field. (**B**) Polished rice grains of 'DHY615'.

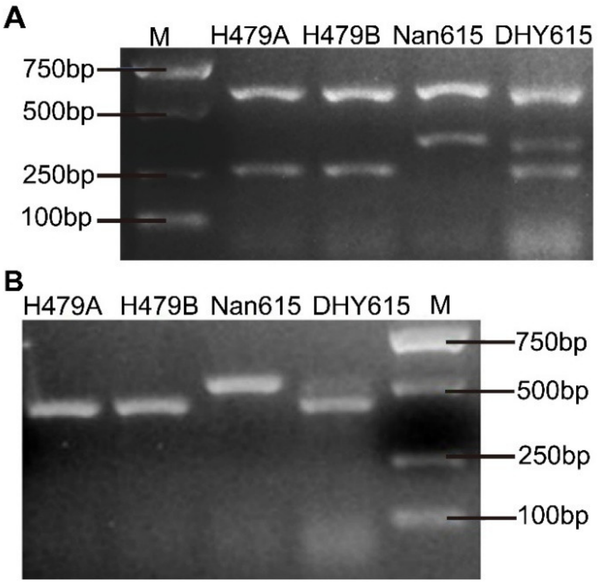

Figure S2. *OsBADH2* and *Rf1a* gene amplification of 'DHY615' and its' parents, the maintainer line H479B. (**A**) *OsBADH2* gene. **(B)** *Rf1a* gene. Note: M is DL2000 DNA Marker.
