# Supplementary figures and images for: Breeding D1-Type Hybrid Japonica Rice in Diverse Upland Rainfed Environments
Source: Int J Mol Sci. 2025 Mar 31;26(7):3246. doi: 10.3390/ijms26073246 (PMC11989851; doi:10.3390/ijms26073246)

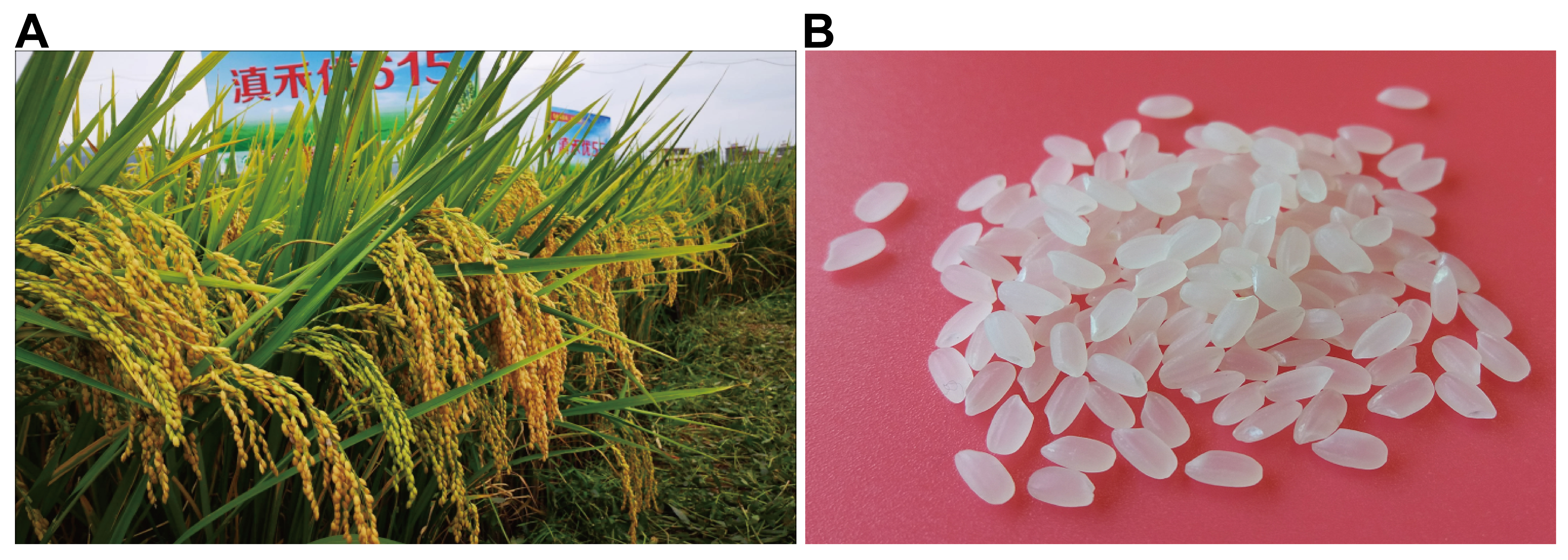

Supplement: Supplementary file 1 [file ijms-26-03246-s001.zip › Finally-Supplementary Materials-IJMS3515233/Figure S1. Plant morphology and polished rice grains of ‘DHY615’..jpg]

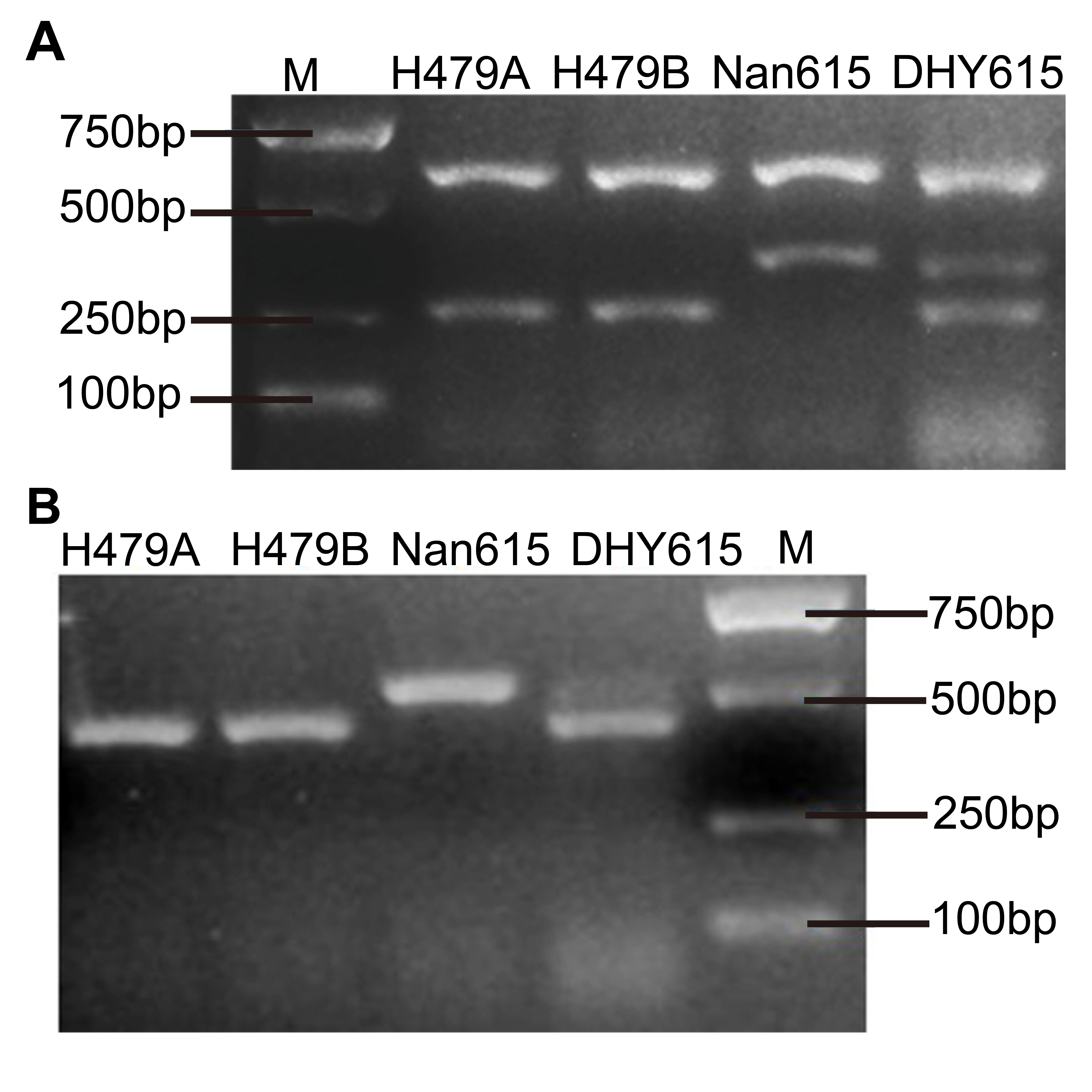

Supplement: Supplementary file 1 [file ijms-26-03246-s001.zip › Finally-Supplementary Materials-IJMS3515233/Figure S2. OsBADH2 and Rf1a gene amplification of ‘DHY615’ and its’ parents, the maintainer line H479B..jpg]
